# GSTM2 alleviates heart failure by inhibiting DNA damage in cardiomyocytes

**DOI:** 10.1186/s13578-023-01168-3

**Published:** 2023-11-30

**Authors:** Hongfei Xu, Zhen Wang, Yalin Wang, Shaobo Pan, Wenting Zhao, Miao Chen, Xiaofan Chen, Tingting Tao, Liang Ma, Yiming Ni, Weidong Li

**Affiliations:** 1 Department of Cardiovascular Surgery, The First Affiliated Hospital of Zhejiang University, School of Medicine, Number 79 Qingchun Road, Hangzhou, China; 2 Department of Operation Room, The First Affiliated Hospital of Zhejiang University, School of Medicine, Hangzhou, China; 3 Department of Cardiology, The First Affiliated Hospital of Zhejiang University, School of Medicine, Hangzhou, China

**Keywords:** Heart failure, Cardiac hypertrophy, Proteomics, Glutathione *S*-transferase M2-2, Inflammation

## Abstract

**Background:**

Heart failure (HF) seriously threatens human health worldwide. However, the pathological mechanisms underlying HF are still not fully clear.

**Results:**

In this study, we performed proteomics and transcriptomics analyses on samples from human HF patients and healthy donors to obtain an overview of the detailed changes in protein and mRNA expression that occur during HF. We found substantial differences in protein expression changes between the atria and ventricles of myocardial tissues from patients with HF. Interestingly, the metabolic state of ventricular tissues was altered in HF samples, and inflammatory pathways were activated in atrial tissues. Through analysis of differentially expressed genes in HF samples, we found that several glutathione *S*-transferase (GST) family members, especially glutathione *S*-transferase M2-2 (GSTM2), were decreased in all the ventricular samples. Furthermore, GSTM2 overexpression effectively relieved the progression of cardiac hypertrophy in a transverse aortic constriction (TAC) surgery-induced HF mouse model. Moreover, we found that GSTM2 attenuated DNA damage and extrachromosomal circular DNA (eccDNA) production in cardiomyocytes, thereby ameliorating interferon-I-stimulated macrophage inflammation in heart tissues.

**Conclusions:**

Our study establishes a proteomic and transcriptomic map of human HF tissues, highlights the functional importance of GSTM2 in HF progression, and provides a novel therapeutic target for HF.

**Supplementary Information:**

The online version contains supplementary material available at 10.1186/s13578-023-01168-3.

## Introduction

Heart failure (HF) is a leading cause of death worldwide, and it is one of the fastest growing health concerns, with a global prevalence of ~ 40 million individuals [1]. However, HF is a complex clinical syndrome with different clinical phenotypes, and it is characterized by a diverse spectrum of structural abnormalities of the left ventricle (LV) [2]. Pathological cardiac hypertrophy is generally considered a critical risk factor for HF [3]. To date, even though numerous advances have been made in HF therapy, there is still a lack of effective drugs [4]. Therefore, elucidating the molecular events that lead to HF is urgently needed in order to identify specific targets for novel drug research and development.

From an anatomical view, heart tissue includes four cavities, four valves, large arteries, and veins, and heart tissue is primarily composed of cardiac fibroblasts (CFs), cardiomyocytes, smooth muscle cells (SMCs), and endothelial cells (ECs) [5, 6]. During the progression of HF, the expression profiles of genes and proteins in different cardiac regions change in different ways [7–9]. Previously, Lau et al. used an integrated omics approach to map the landscape of proteomic remodeling in a pathological model of cardiac hypertrophy in inbred mice from six divers, and they identified 273 candidate disease signatures in 36 nonredundant cardiac pathways that were reproducibly altered, providing important insights into disease pathogenesis in vivo [10]*.* Recently, Doll et al. established a region- and cell type-resolved quantitative proteomic map of the normal human heart, and they identified proteomic differences in different heart regions, suggesting functional differences, and identifying potential cell type markers [11]. However, to date, a detailed global protein expression “footprint” in different regions of human HF tissues does not exist.

In this study, we performed proteomics and transcriptomics analyses on samples from human HF patients and healthy donors to provide an overview of the detailed changes in proteins and mRNAs expression that occur in HF. We found that there were substantial differences in the protein expression changes in the atria and ventricles of the myocardium from patients with HF. The metabolic state in ventricular tissues from HF patients was altered, and inflammatory pathways were activated in atrial tissues. Interestingly, glutathione *S*-transferase M2-2 (GSTM2) was significantly decreased in human and mouse HF tissues. Overexpression of GSTM2 attenuated DNA damage and extrachromosomal circular DNA (eccDNA) production in cardiomyocytes, thereby alleviating interferon-I-stimulated macrophage activation and inflammation in heart tissues. Our study highlights the functional importance of GSTM2 in HF progression and provides a novel therapeutic target for HF.

## Methods and materials

### Heart samples

Forty HF tissue samples from eight HF patients and three healthy heart tissue samples from three donors were collected and used in this study. All HF patients were diagnosed with dilated cardiomyopathy with EF < 25% (systolic HF), and the clinical details of the HF patients are shown in Additional file 1: Table S1. All HF patients were diagnosed with HF (Function Capacity IV, Objective Assessment D, based on New York Heart Association functional classification) at least 3 months before heart transplantation. Written informed consent was obtained from the patients. Samples were collected in accordance with the human research protocol that was approved by the Research Ethics Committee of the First Affiliated Hospital, College of Medicine, Zhejiang University. The forty-three heart tissue samples were collected from the following regions: eight left atrium (LA) samples from HF patients, eight LV samples from HF patients, eight right atrium (RA) samples from HF patients, eight right ventricle (RV) samples from HF patients, eight Interventricular septum (IVS) samples from HF patients, and three control left atrium (CLA/C_LA) samples from healthy donors. These samples were explanted by an official medicolegal expert. The samples were stored at − 80 °C after collection.

### Proteomics analysis

Forty-three heart samples were used to perform label-free quantitative proteomics at Novogene Co., Ltd. (Beijing, China), as previously described [12–14]. Briefly, the samples were washed three times with cold PBS, homogenized in liquid nitrogen, and sonicated three times on ice. Then, the supernatants were collected after centrifugation, reduced by incubation with 5 mM dithiothreitol for 30 min at 56 °C and alkylated by incubation with 11 mM iodoacetamide for 15 min at room temperature in the dark. Afterword, the samples were diluted by adding 100 mM triethylammonium bicarbonate to urea at a concentration of less than 2 M, digested by incubation with the mixture at a 1:50 trypsin-to-protein mass ratio overnight, and then digested by incubation with a 1:100 trypsin-to-protein mass ratio for 4 h. The components that were obtained by high pH reversed-phase separation were redissolved in 20 µl of 2% methanol and 0.1% formic acid solution. The sandwich method was used, and 10 µl of peptide samples were separated using a C18 column with a flow rate of 350 nl/min. The isolated peptides were detected by mass spectrometry. The mass spectral data were analyzed and screened by MaxQuant software, and the identified differentially expressed proteins were matched by searching the UniProt Human database. The default cutoff value of a twofold change was used, and a p value < 0.05 was considered to indicate a significant difference.

### Transcriptomics analysis

Forty-three heart samples were used to perform transcriptomics analysis at the Novogene Co., Ltd. (Beijing, China). Briefly, the total RNA was isolated using RNeasy kit (Qiagen China (Shanghai) Co Ltd, Shanghai, China), and the quality was analyzed using an Agilent 2100 bioanalyzer (Agilent technologies, California, CA, USA). The gene expression profiles were investigated using Illumina HiSeq2000 RNA Sequencing (Illumina, San Diego, CA, USA).

### Bioinformatics analysis

Kyoto Encyclopedia of Genes and Genomes (KEGG) analysis of the significantly upregulated or downregulated proteins or mRNAs (p value < 0.05 and | log2 FoldChange (FC) |> 1.2 or < 0.44) was performed with the “clusterProfiler” package for functional enrichment [15]. KEGG analysis results that had an adjusted p values of 0.05 were considered statistically significant. For hierarchical clustering, protein abundances were clustered using Euclidean as a distance measure for column and row clustering. Weighted gene coexpression network analysis (WGCNA) was used to perform module identification and the division of proteomes or transcriptomes [16], according to correlations with different regions of the heart or clinical features. The soft threshold power value was set to 9 for subsequent analyses. A topological overlap matrix (TOM) was used to measure the connection strength between genes (Dynamic Tree Cut: minModuleSize = 50; Cluster Cut: MEDissThres = 0.25). Uniform Manifold Approximation and Projection (UMAP) was used for the visualization of cell clusters. Clustering was performed to obtain integrated expression values based on shared-nearest-neighbor (SNN) graph clustering (Louvain community detection-based method) using “FindClusters” with a resolution of 0.8. Sankey analysis was performed using the “ggalluvial” package of R software. Mean log2 ratios of biological triplicates and the corresponding p values were visualized with volcano plots.

Spearman correlation coefficients were used to measure the correlation between mRNA expression and protein abundance for each gene‒protein pair across all 43 heart samples. In addition, the p value that corresponded to the correlation coefficient was calculated and adjusted by FDR correction. The significance of the correlation pair was determined based on an adjusted p value cutoff of 0.01. Then, the mRNA‒protein matches were determined with a median Spearman correlation of r = 0.54. Moreover, mRNA and protein expression levels were positively correlated for most (98.6%) mRNA‒protein pairs, and 90.3% showed a significant positive correlation (multiple test adjusted p value < 0.01).

### Single-cell RNA sequencing (scRNA-seq) analysis

Single cell RNA sequence data from human HF tissues (LA + LV) were reanalyzed using the FindVariableFeatures function, and downstream procedures were performed using the ScaleData and runPCA functions as previously described [17, 18].

### Transverse aortic constriction (TAC) mouse model

All the animal experimental procedures were approved by the Animal Care Ethics Committee of the First Affiliated Hospital Zhejiang University School of Medicine, and the experiments were performed in compliance with the “Guide for the Care and Use of Laboratory Animals” from the US National Institutes of Health. Eight-week-old male C57BL/6J mice were purchased from the Model Animal Research Center of Nanjing University and housed in the Laboratory Animals Center of the First Affiliated Hospital of Zhejiang University School of Medicine under conditions of controlled temperature and humidity. HF was established in the mice by TAC as previously described [17, 19]. Briefly, mice were anesthetized via the intraperitoneal administration of 0.3% sodium pentobarbital (75 mg·kg^−1^) intraperitoneally, and the chest cavity was opened to expose the aortic arch. Then, the aortic arch was tied with a 6–0 nylon suture between the brachiocephalic and left common arteries with a homemade L-shaped 26G cushion needle. After ligation, the needle was quickly removed, causing approximately 70% contraction, and the skin was closed. The sham operation followed an identical procedure, except that the thread was not ligated. Moreover, the mice were injected with rAAV9 (4 × 10^11^ vector genomes (vg)/mouse) carrying an empty vector, or GSTM2 via the tail vein.

### Primary cardiomyocyte isolation and culture

Primary cardiomyocytes were isolated from neonatal mice (1–2 days) as previously described [17]. To induce hypertrophy, the cardiomyocytes were treated with phenylephrine (PE, 50 μmol/l, Sigma, USA) for 24 h.

### Echocardiographic evaluation

Echocardiographic evaluation was performed as previously described [17]. The left ventricular (LV) end-diastolic diameter (LVEDd) and LV ejection fraction (EF%) were measured from the LV M-mode at the mid-papillary muscle level. The mice were euthanized by cervical dislocation after echocardiographic evaluation at 4 weeks after the operation. Mouse hearts were dissected and weighed or measured to compare the heart weight (HW)/body weight (BW) ratios.

### Histological study

The heart tissue sections were embedded in paraffin and cut into 5-μm serial sections and then stained with a hematoxylin and eosin (HE) staining kit (Byotime, China) to assess pathological changes in the myocardium, and a Masson staining kit (SbjBio, China) was used to evaluate cardiac fibrosis according to the manufacture’s instructions.

### Immunofluorescence

Heart tissue sections were blocked with goat serum at room temperature for 30 min, incubated with primary antibodies against α-actinin (1:200, A7811, Sigma, USA) and GSTM2 (1:200, Ab196503, Abcam, USA) at 4 °C overnight, and treated with fluorescently labeled secondary antibodies for 1 h at 37 °C. The nuclei were counterstained with DAPI. To evaluate cardiomyocyte size, the heart tissue sections were incubated with fluorescein-conjugated wheat germ agglutinin stain (1:200, Alexa Fluor‐488, Invitrogen, CA), and approximately 50–100 randomly chosen cardiomyocytes were analyzed by using ImageJ software to measure the cross-sectional cardiomyocyte area. To analyze DNA double-stranded breaks in cardiomyocytes, the cardiomyocytes were incubated with antibodies against histone family member X (H2AX) that was phosphorylated at the Ser 139 site (γ-H2AX) (1:200, Ab1174, Abcam, USA) and then stained with a fluorescently labeled secondary antibody for 1 h at 37 °C. After washing, the cells were imaged.

### Quantitative real-time PCR (qRT‒PCR) and enzyme-linked immunosorbent assay (ELISA)

After infection with AAV9-GSTM2 or AAV9-Control for 48 h and then treatment with or without PE for 24 h, the cell culture supernatants were collected to measure the protein levels of IFN-α and IFN-β using the Mouse Interferon alpha 1 ELISA Kit (ab252352, Abcam) and Mouse IFN beta ELISA Kit (ab252363, Abcam), respectively. To further explore the effect of cardiomyocytes on macrophages, cell culture supernatants from treated cardiomyocytes were incubated with RAW264.7 macrophage cultures for 24 h. Then, total RNA was extracted with TRIzol reagent (Invitrogen, USA), and reverse-transcribed into cDNA using PrimeScript RT Master Mix (Takara, Japan). A SYBR Premix Ex TaqII (Takara, Japan) kit was used for qRT‒PCR with an ABI PRISM 7500 Detection System (ABI, USA). Relative expression values were normalized to GAPDH expression. Lentivirus was purchased from Gene-Pharma Company (Shanghai, China). The primers that were used were as follows: interleukin-6 (IL-6), F 5ʹ-TCTATACCACTTCACAAGTCGGA-3ʹ, and R 5ʹ-GAATTGCCATTGCACAACTCTTT-3ʹ; tumor necrosis factor-α (TNF-α), F 5ʹ-CTGAACTTCGGGGTGATCGG-3ʹ, and R 5ʹ-GGCTTGTCACTCGAATTTTGAGA-3ʹ; interferon-stimulated gene 15 (ISG15), F 5ʹ-GGTGTCCGTGACTAACTCCAT-3ʹ, and R 5ʹ-TGGAAAGGGTAAGACCGTCCT-3ʹ; myxoma resistance 1 (Mx1), F 5ʹ-GACCATAGGGGTCTTGACCAA-3ʹ, and R 5ʹ-AGACTTGCTCTTTCTGAAAAGCC-3ʹ; chemokine (C-X-C motif) ligand 10 (Cxcl10), F 5ʹ-CCAAGTGCTGCCGTCATTTTC-3ʹ, and R 5ʹ-GGCTCGCAGGGATGATTTCAA-3ʹ; and GAPDH, F 5ʹ-TGGATTTGGACGCATTGGTC-3ʹ, and R 5ʹ-TTTGCACTGGTACGTGTTGAT-3ʹ.

### Western blotting

Western blotting was performed as previously described [17]. Primary antibodies against IRF3 (1:1000, 11312-1-AP, Proteintech), STAT1 (1:1000, 66545-1-Ig, Proteintech), phosphorylated IRF3 (pIRF3) (1:1000, ab76493, Abcam), phosphorylated STAT1 (pSTAT1) (1:1000, ab215820, Abcam) and GAPDH (1:1000, ab8245, Abcam, USA) were used.

### Statistical analysis

All the statistical analyses were performed with GraphPad Prism 7.0. The data are expressed as the mean ± standard deviation (SD). Differences between two groups were analyzed by using unpaired Student’s t test. Furthermore, differences among multiple groups were analyzed by using two-way ANOVA. Differences were considered statistically significant when p < 0.05.

## Results

### Proteomics analysis of human HF samples

**Fig. 1:**
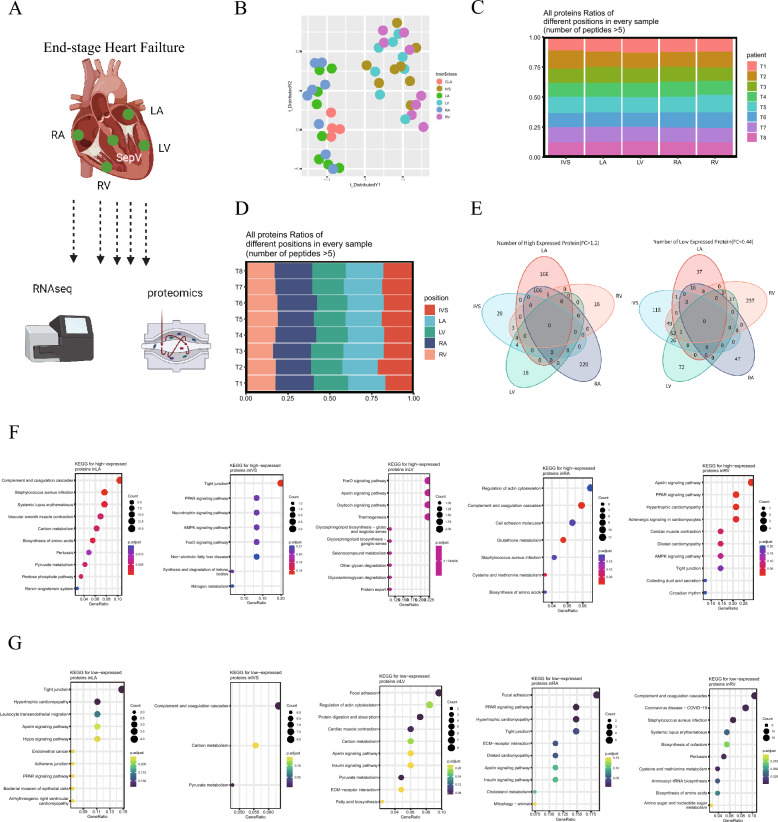
Proteomics analysis of samples from human heart failure patients. **A** Schematic depiction of the experimental design. **B** t-distributed stochastic neighbor embedding (tSNE) analysis of the 43 heart samples based on their proteomic expression profiles. **C**, **D** All protein ratios of different positions in every sample. **E** Commonly and specifically expressed proteins in five heart regions. **F** KEGG analysis of the proteins that were specifically upregulated in five different heart regions. **G** KEGG analysis of the proteins that were specifically downregulated in five different heart regions

To obtain an overview of the detailed changes in protein and mRNA expression that occur in HF, we collected a total of 43 samples from 8 HF samples and 3 healthy donors, including 8 LA samples, 8 LV samples, 8 RA samples, 8 RV samples, 8 IVS samples, and 3 healthy CLA samples, for proteomics and transcriptomics analysis. A total of 5311 proteins were identified in all the heart regions (Fig. 1A). We then assessed the proteomic similarities and differences in the 43 heart regions with t-distributed stochastic neighbor embedding (tSNE) analysis and found that the atrial (CLA, LA and RA) and ventricular (IVS, RV and LV) regions were clearly clustered (Fig. 1B). Furthermore, the expression of the identified proteins in different heart regions from different samples were similar (Fig. 1C and D, and Additional file 2: Figure S1A and B), suggesting unbiased results of proteomics analysis. Moreover, the significantly differentially expressed proteins in different regions of the HF samples intersected with each other, and 166 s proteins that were specifically upregulated (p < 0.05 and FC > 1.2) in the LA, 16 proteins in the RV, 220 proteins in the RA, 18 proteins in the LV and 20 proteins in the IVS were identified, respectively (Fig. 1E). Thirty-seven proteins that were specifically downregulated (p < 0.05 and FC < 0.44) in the LA, 237 proteins in the RV, 47 proteins in the RA, 72 proteins in the LV and 118 proteins in the IVS were identified (Fig. 1E). As expected, heart tissue markers, such as titin (TTN) [20], myosin heavy chain 7 (MYH7) [17], plectin (PLEC) [21] and desmoplakin (DSP) [22], were enriched in all the samples (Additional file 2: Figure S1C and D). TXNRD1, ADH1B, CD14, GCLC, NPPA, and NPPB were more enriched in the atrial tissues, while SRP54, COX20, PLIN5, MAP3K5, PFKFB2 and SLC12A7 were more enriched in the ventricular tissues (Additional file 2: Figure S1E), suggesting differential metabolism and inflammation states between these tissues. QPCR analysis also yielded consistent results (Additional file 2: Figure S1F and G). Moreover, KEGG analysis showed that the proteins that were specifically upregulated in the LA were enriched in the pyruvate metabolism and pentose phosphate pathway, those in the IVS were enriched in the tight junction, nitrogen metabolism and peroxisome proliferator-activated receptors (PPAR) signaling pathway, those in the LV were enriched in the forkhead box protein O (FoxO) signaling pathway and apelin signaling pathway, those in the RA were enriched in the regulation of glutathione metabolism and cysteine and methionine metabolism, and those in the RV were enriched in the apelin signaling pathway and PPAR signaling pathway (Fig. 1F). In addition, the KEGG analysis showed that the proteins that were specifically downregulated in the LA were enriched in tight junction and hypertrophic cardiomyopathy, those in the IVS were enriched in pyruvate metabolism and carbon metabolism, those in the LV were enriched in pyruvate metabolism and carbon metabolism, those in the RA were enriched in focal adhesion and the PPAR signaling pathway, and those in the RV were enriched in biosynthesis of amino acids and cysteine and methionine metabolism (Fig. 1G). Interestingly, the metabolic pathways (pyruvate metabolism and carbon metabolism) that were enriched in atrial tissues were not enriched in ventricular tissues. However, PPAR signaling, which was enriched in ventricular tissues, was lacking in atrial tissues.

### Identification of key modules based on WGCNA

**Fig. 2:**
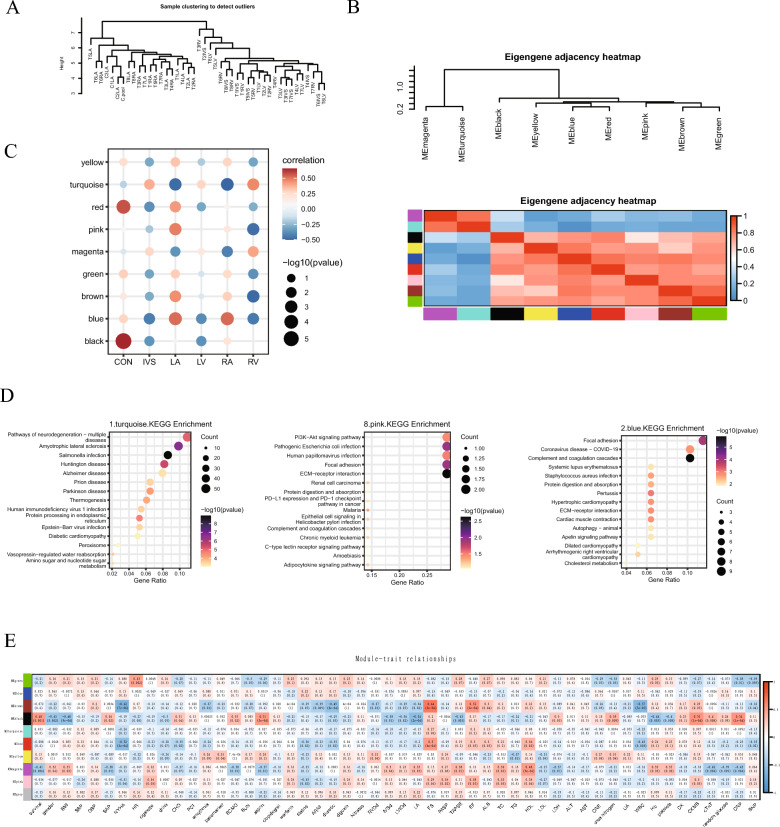
Identification of key modules based on WGCNA. **A** Unsupervised hierarchical clustering analysis of the identified proteins with significantly different expression across the samples (FDR < 0.05). **B** Coexpression analysis by WGCNA. **C** Correlation analysis of the different modules and different heart regions. **D** KEGG analysis of the proteins in the indicated modules. **E** Correlation analysis between clinical phenotypes and modules

Next, we further performed unsupervised hierarchical clustering of the identified proteins that exhibited significantly different expression across the samples (FDR < 0.05). As expected, atrial samples were more similar than ventricular samples indicating that atrial and ventricular tissues had different protein expression patterns (Fig. 2A). Furthermore, the proteins could be clustered into nine modules, and module MEmagenta had high similarity to module MEturquoise. The other seven modules had high similarity (Fig. 2B), as determined by WGCNA, which is a method for analyzing the gene expression patterns of multiple samples that can be used to cluster genes and form modules according to similar gene expression patterns and analyze the relationship between modules and specific features. Correlation analysis of the nine modules and different heart regions showed that turquoise (module MEturquoise)-related proteins were lacking in atrial samples (LA and RA) but were enriched in ventricular samples (IVS, LV and RV). Blue (module MEblue)-related proteins showed the opposite trends (Fig. 2C). Moreover, the KEGG analysis showed that proteins in the turquoise module were mainly enriched in peroxisome, diabetic cardiomyopathy, and amino sugar and nucleotide sugar metabolism, suggesting changes in the metabolic state of ventricular tissue. The proteins in blue module were enriched in COVID-19, systemic lupus erythematosus (SLE) and *Staphylococcus aureus* (SA) infection, suggesting that inflammatory pathways were activated in atrial tissues (Fig. 2D). These results indicated that there was a great difference in the change in protein expression pattern between the atria and ventricles of the myocardium from patients with HF. Amino acid metabolism-related proteins were upregulated in the ventricular myocardium, while extracellular matrix, cell adhesion and cholesterol metabolism-related proteins were downregulated. The changes in the atrium exhibited the opposite trends. In addition, correlation analysis of the nine modules and patient clinical features showed that the green module was positively associated with heart rate, and the proteins in the turquoise module were mainly enriched in glycolysis, carbon metabolism, and the hypoxia-inducible factor (HIF)-1 signaling pathway. The brown module was positively associated with the short-axis shortening rate (FS) and ejection fraction (EF), and the proteins in the brown module were mainly enriched in the complement and coagulation cascades (Fig. 2E).

### Identification of key modules for transcriptomics based on WGCNA

To obtain a functional view of the transcriptomic differences among human HF samples, unsupervised hierarchical clustering of the identified genes that were significantly differentially expressed in different heart regions (FDR < 0.05) was used to cluster individuals (Additional file 2: Figure S2A). Coexpression analysis by WGCNA showed that the transcripts were clustered into nine modules; MEgreen, MEblue and MEbrown had high similarity, and the other six modules had a high similarity (Additional file 2: Figure S2B). Correlation analysis of the nine modules and different heart regions showed that the red module was enriched in the RA and lacking in the LV, and the transcripts in the red module were mainly enriched in vascular smooth muscle contraction, and the focal adhesion signaling pathway (Additional file 2: Figure S2C). The green module was enriched in the RV and lacking in the LA, and the transcripts in the green module were mainly enriched in carbon metabolism, the TCA cycle, diabetic cardiomyopathy, pyruvate metabolism, and fatty acid metabolism (Additional file 2: Figure S2C). Correlation analysis of the nine modules and patient clinical features showed that the black module was positively associated with creatine kinase (CK), alanine aminotransferase (ALT), aspartate aminotransferase (AST), lactate dehydrogenase (LDH), clopidogrel administration, and a previous medical history of percutaneous coronary intervention (PCI), and it was negatively associated with diuretic administration (Additional file 2: Figure S2D).

### Multiomics analysis of HF disease signatures

**Fig. 3:**
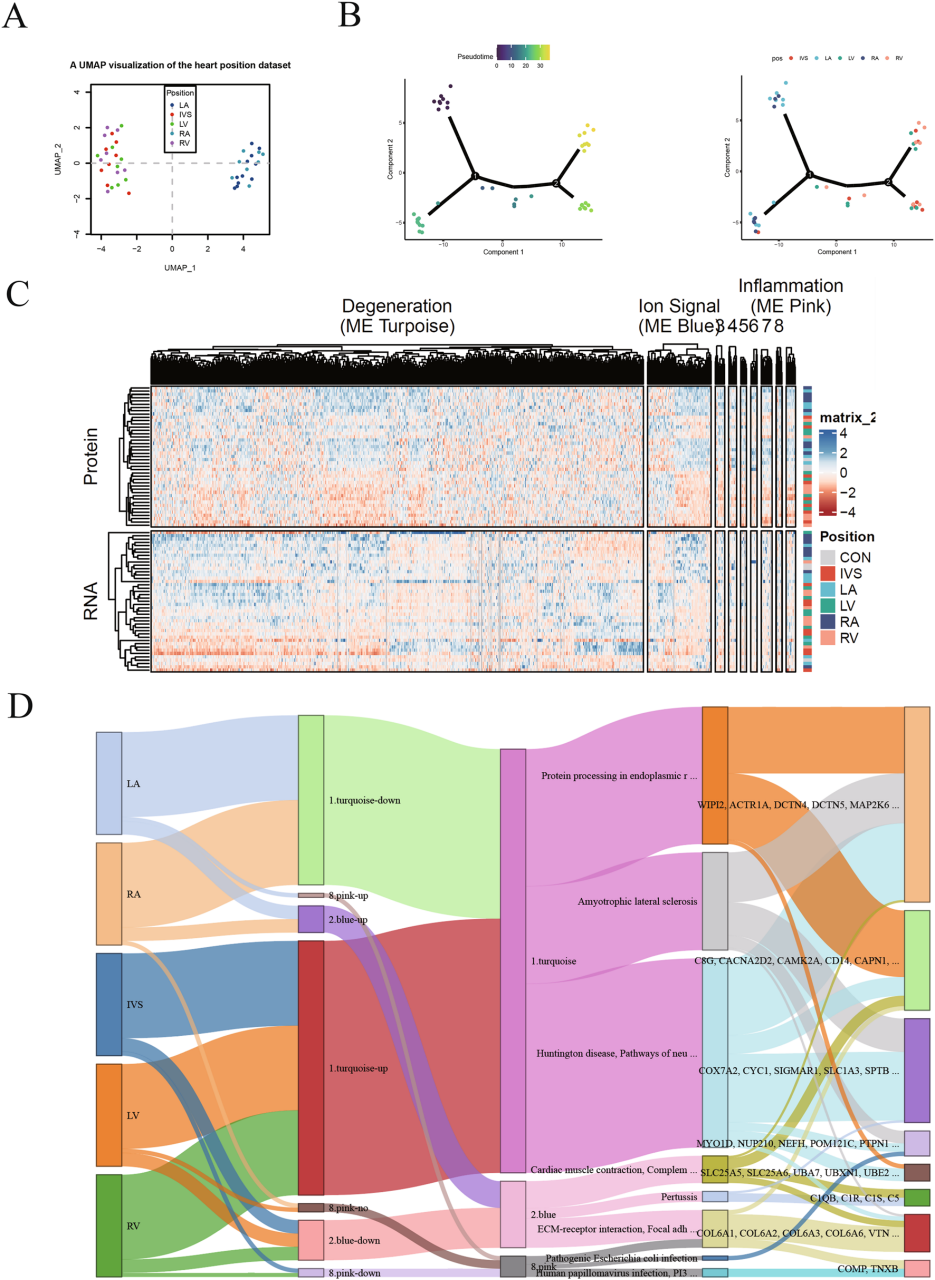
Multiomics analysis of disease signatures of heart failure. **A** Principal component analysis (PCA) of the 40 samples from heart failure patients based on their proteomic expression profiles. **B** Pseudotime series analysis of the correlation between protein/RNA profiles and heart regions. **C** WGCNA of the correlation between protein expression and RNA transcription in the indicated three modules. **D** Sankey analysis of heart regions, modules, signaling pathways, and proteins/RNAs

Subsequently, we integrated proteomics and transcriptomics data to examine the combined contribution to HF, and a subset of genes/proteins that were commonly observed in the different anatomical regions was selected for further analysis. Principal component analysis (PCA) showed that the genes/proteins were separated into two clusters, mainly an atrial cluster (LA and RA) and a ventricular cluster (IVS, RV and LV) (Fig. 3A). Furthermore, the pseudotime series analysis showed that the correlation between protein and RNA expression was stronger in the LA and RA regions than in the RV, LV and IVS regions, suggesting that different expression patterns in atrial and ventricular tissues during HF (Fig. 3B). Moreover, WGCNA showed that the correlation between protein expression and RNA transcription was strongest in the turquoise module (degeneration pathway), blue module (ion signal) and pink module (inflammation) (Fig. 3C), suggesting that these biological activities were similarly regulated in different cardiac regions. Interestingly, Sankey analysis showed that the downregulated proteins/RNAs in the LA and RA regions, and the upregulated proteins/RNAs in the RV, LV and IVS regions were all enriched in the turquoise modules, and these proteins/RNAs were involved in protein processing in the endoplasmic reticulum, amyotrophic lateral sclerosis and Huntington disease (Fig. 3D).

### Analysis of the differentially expressed genes in HF samples

**Fig. 4:**
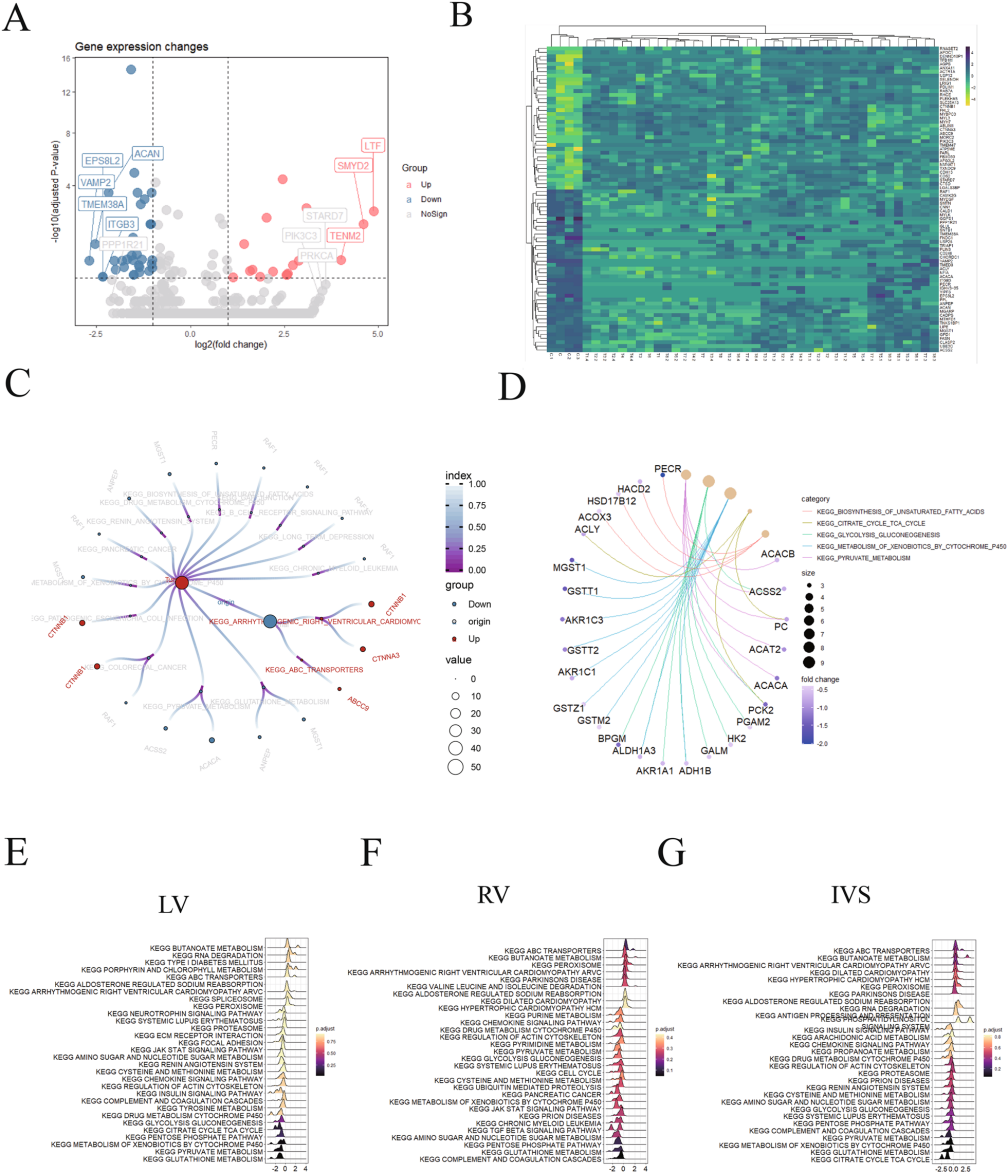
Analysis of the differentially expressed genes in samples from heart failure patients. **A** Volcano plot of the differentially expressed genes in samples from heart failure patients. **B** Heatmap of the differentially expressed genes in different heart samples. **C** and **D** KEGG analysis of the differentially expressed genes. **E**, **F** and **G** KEGG analysis of the differentially expressed genes in the LV, RV and IVS, respectively

Next, we analyzed the differentially expressed genes in the HF samples (LA + LV + RA + RV + IVS) compared to the normal atrial tissues (CLA). As shown in Fig. 4A and B, 37 significantly upregulated genes and 42 significantly downregulated genes were identified. Furthermore, KEGG analysis showed that Catenin beta1 (CTNNB1) was significantly upregulated, and acetyl-CoA carboxylase 1 (ACACA), Rubisco accumulation factor 1 (RAF1), peroxisomal trans-2-enoyl-CoA reductase (PECR) and microsomal glutathione transferase (MGST) were notably downregulated (Fig. 4C). Moreover, we found that several glutathione *S*-transferase (GST)-related genes, including MGST1, glutathione *S*-transferase T1 (GSTT1), glutathione transferase zeta1 (GSTZ1), and glutathione *S*-transferase M2-2 (GSTM2), were identified among the most downregulated signaling pathways (Fig. 4D). Finally, KEGG analysis of the differentially expressed genes in the LV, RV and IVS was performed. As shown in Fig. 4E–G, most pathways that were enriched in the LV, RV and IVS regions were similar. Interestingly, the antigen processing and presentation pathway was enriched in the IVS regions, and energy metabolic pathways were more enriched in the LV and RV regions, while GST family members were lacking in all ventricular samples, suggesting that oxidative stress was imbalanced in cardiomyocytes.

### GSTM2 is reduced in hypertrophic heart tissues

**Fig. 5:**
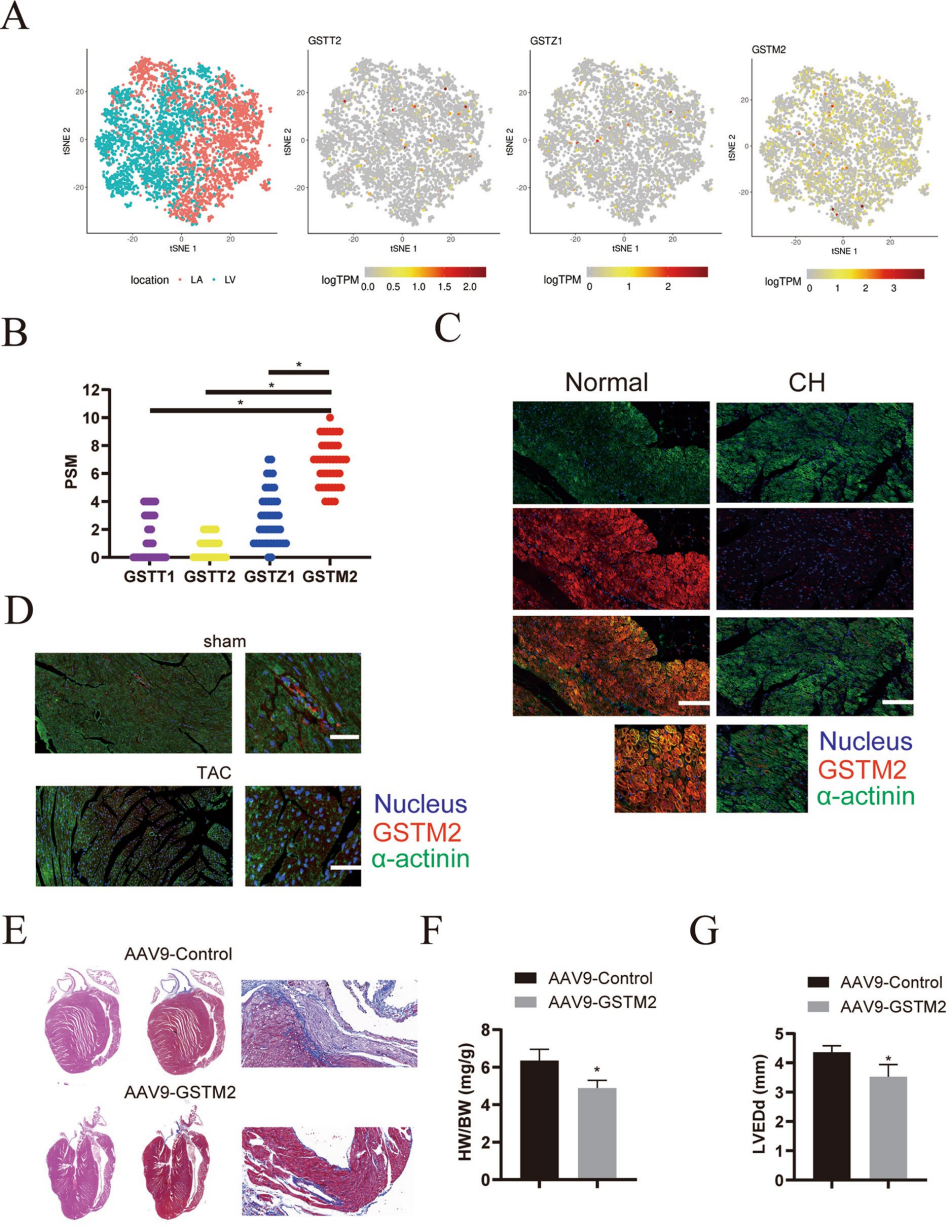
Analysis of the differentially expressed genes in samples from heart failure patients. **A** 2D visualization of GSTT2, GSTZ1 and GSTM2 gene expression in the LV and LA. **B** Analysis of GSTT2, GSTZ1 and GSTM2 protein abundances according to proteomics. **C** Immunofluorescence analysis of GSTM2 (red) expression in cardiomyocytes (stained with the cardiomyocyte marker α-actinin, green) from clinical human cardiac hypertrophy (CH) tissues and normal heart tissues. The nuclei were stained with DAPI (blue). Scale bar: 200 μm. **D** Immunofluorescence analysis of GSTM2 (red) expression in cardiomyocytes (stained with the cardiomyocyte marker α-actinin, green) from the heart tissues of sham or TAC model mice. The nuclei were stained with DAPI (blue). Scale bar: 100 μm. **E** Representative Masson staining results to assess fibrosis in heart tissues from mice injected with AAV9-GSTM2 or control AAV9 via the tail vein for 3 weeks, then subjected to TAC, and analyzed 4 weeks later. **F** Statistical analysis of the heart weight (HW)/body weight (BW) ratios of mice subjected to TAC surgery 4 weeks after surgery (n = 6 mice per group; *p < 0.05 versus sham group). **G** Assessment of left ventricular end-diastolic diameter (LVEDd) in mice, n = 6. ^*^p < 0.05

First, we measured the expression of GSTT2, GSTZ1 and GSTM2 in human cardiomyocytes using published scRNA-seq data [18]. As shown in Fig. 5A, GSTM2 was more highly enriched than GSTZ1 and GSTT2. Consistent with the scRNA-seq data, our proteomics analysis indicated that GSTM2 was the most highly expressed GST (Fig. 5B). Moreover, we also measured the mRNA and protein levels of GSTM2 and GSTZ1 in clinical samples and obtained consistent results (Additional file 2: Figure S3A and B). However, GSTM2 was reduced in hypertrophic heart tissues from both humans and mice (Fig. 5C and D, and Additional file 2: Figure S3D). We suspected that GSTM2 participated in cardiac hypertrophy progression. To test our hypothesis, a GSTM2 overexpression vector (AAV9-GSTM2) was administered to the hearts of TAC model mice by tail vein injection (Additional file 2: Figure S3C). GSTM2 overexpression indeed partly reversed TAC-induced cardiac hypertrophy (Additional file 2: Figure S3E and F) and collagen deposition (Fig. 5E). In addition, heart function, including HW/BW, LVEDd and EF, was improved after AAV9-GSTM2 delivery (Fig. 5F, G and Additional file 2: Figure S3G and H). These results indicate that GSTM2 mitigates cardiac hypertrophy progression.

### GSTM2 attenuates DNA damage in hypertrophic heart tissues

**Fig. 6:**
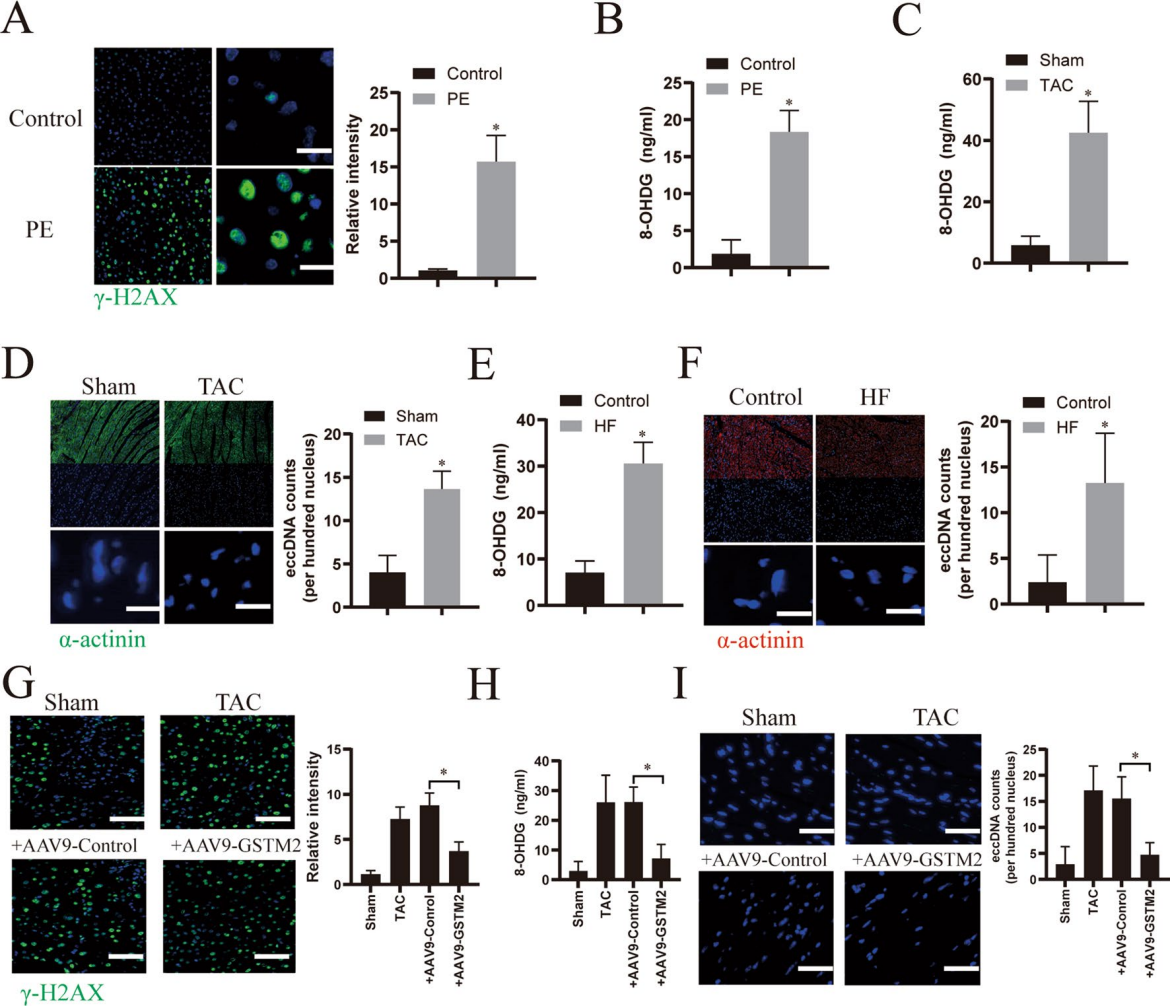
GSTM2 attenuates DNA damage in hypertrophic heart tissues. **A** Immunofluorescence analysis of γ-H2AX (green) in primary cardiomyocytes stimulated with phenylephrine (PE, 50 μmol/l), or DMSO (as a control) for 24 h. Nuclei were stained with DAPI (blue). Scale bar: 50 μm. **B** Assessment of the 8-OHDG contents in the culture supernatants from PE- or DMSO (as a control)-treated cardiomyocytes. n = 6. ^*^p < 0.05. **C** Assessment of the 8-OHDG contents in the heart tissues of sham or TAC model mice. n = 6. ^*^p < 0.05. **D** Representative confocal immunofluorescence images of cardiomyocytes (α-actinin, green) from heart tissues of sham or TAC model mice. The nuclei were stained with DAPI (blue). eccDNA was quantified by analyzing the intensity of DAPI outside nuclei. ^*^p < 0.05. Scale bar: 100 μm. **E** Assessment of the 8-OHDG contents in clinical human heart failure (HF) tissues and normal heart tissues (as control). n = 3. ^*^p < 0.05. **F** Representative confocal immunofluorescence images of cardiomyocytes (α-actinin, red) from clinical human HF tissues and normal heart tissues (as control). The nuclei were stained with DAPI (blue). eccDNA was quantified by analyzing the intensity of DAPI outside the nuclei. ^*^p < 0.05. Scale bar: 100 μm. **G** Immunofluorescence analysis of γ-H2AX (green) in primary cardiomyocytes infected with AAV9-GSTM2 or control AAV9 for 48 h and then treated with PE for 24 h. Scale bar: 200 μm. **H** Assessment of the 8-OHDG content in the culture supernatants from cardiomyocytes infected with AAV9-GSTM2 or control AAV9 for 48 h and then treated with PE for 24 h. n = 6. ^*^p < 0.05. **I** Representative confocal immunofluorescence images of cardiomyocytes (α-actinin, green) from the heart tissues of TAC model mice infected with AAV9-GSTM2 or control AAV9. The nuclei were stained with DAPI (blue). eccDNA was quantified by analyzing the intensity of DAPI outside the nuclei. ^*^p < 0.05. Scale bar: 100 μm

As GST family members mainly sustain oxidative stress homeostasis to alleviate DNA damage [23], we further determined the effect of GSTM2 on DNA damage in in vitro and in vivo models of HF. As shown in Fig. 6A and B, DNA damage was notably induced in cardiomyocytes after PE stimulation, as evidenced by the detection of γ-H2AX (a marker of DNA damage) [23] expression and 8-OHDG (a metabolite of DNA damage) [24] contents. Further, 8-OHDG was also significantly increased in the TAC model mice HF samples and human HF samples (Fig. 6C and E). Interestingly, the levels of extrachromosomal circular DNA elements (eccDNAs), which are always released during DNA damage [25, 26], were also increased in hypertrophic heart tissues (Fig. 6D and F). However, overexpression of GSTM2 by AAV9-GSTM2 infection notably alleviated DNA damage, as evidenced by decreased γ-H2AX expression in PE-treated cardiomyocytes (Fig. 6G) and inhibiting of 8-OHDG production (Fig. 6H) and eccDNA release in mouse HF samples (Fig.  6I). Collectively, these data indicate that GSTM2 attenuates DNA damage in hypertrophic heart tissues.

### GSTM2 alleviates IFN-I-stimulated macrophage inflammation

**Fig. 7:**
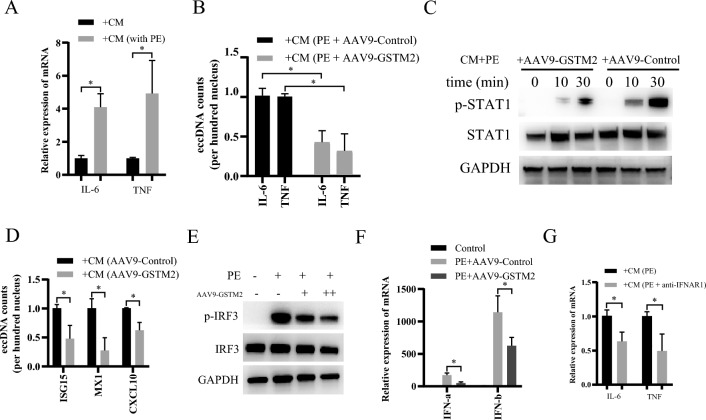
GSTM2 alleviates IFN-I-stimulated macrophage inflammation. **A** qRT‒PCR analysis of IL-6 and TNF-α mRNA expression in RAW264.7 macrophages treated with culture supernatants from PE- or DMSO-treated cardiomyocytes. n = 3. ^*^p < 0.05. **B** qRT‒PCR analysis of IL-6 and TNF-α mRNA expression in the RAW264.7 macrophages treated with culture supernatants from PE-treated cardiomyocytes infected with AAV9-GSTM2 or control AAV9. n = 3. ^*^p < 0.05. **C** Western blotting analysis of STAT1 and p-STAT1 expression in RAW264.7 macrophages treated with culture supernatants from PE-treated cardiomyocytes infected with AAV9-GSTM2 or control AAV9. GAPDH was used as a loading control. **D** qRT‒PCR analysis of ISG15, MX1, and CXCL10 mRNA expression in RAW264.7 macrophages treated with culture supernatants from PE-treated cardiomyocytes infected with AAV9-GSTM2 or control AAV9. n = 3. ^*^p < 0.05. **E** Western blotting analysis of IRF3 and p-IRF3 expression in the cardiomyocytes treated with PE alone or combined with AAV9-GSTM2 infection. GAPDH was used as a loading control. **F** Assessment of IFN-α and IFN-β levels in the culture supernatants from PE-treated cardiomyocytes infected with AAV9-GSTM2 or control AAV9. n = 3. ^*^p < 0.05. **G** qRT-PCR analysis of IL-6 and TNF-α mRNA expression in RAW264.7 macrophages treated with culture supernatants from PE- and IFNAR1 antibody-treated cardiomyocytes. n = 3. ^*^p < 0.05

eccDNA is a potent innate immunostimulant that can generally trigger type I interferon (IFN-I) production by activating cGAS-Sting-IRF3 signaling [25]. We further investigated whether injured cardiomyocytes could affect macrophage inflammation, and we found that culture supernatants from PE-treated cardiomyocytes significantly promoted IL-6 and TNF-α transcription in macrophages (Fig. 7A). However, pretreatment of cardiomyocytes with AAV9-GSTM2 reversed this effect (Fig. 7B). IFN-Is are essential cytokines for macrophage-mediated inflammatory effects, and all the effects of IFN-Is are mediated by IFN-stimulated genes (ISGs), such as ISG15, Mx1 and CXCL10; the expression of these genes is induced through type I interferon receptor subunit 1 (IFNAR1), followed by the activation of the Janus kinase (JAK)-signal transducer and activator of transcription (STAT) signaling [27]. Furthermore, we found that the addition of culture supernatants from PE-treated GSTM2-overexpressing cardiomyocytes notably inhibited STAT1 phosphorylation (Fig. 7C) and ISG expressions (Fig. 7D). Moreover, consistent with the finding that eccDNA triggers IFN-I production by activating cGAS-Sting-IRF3 signaling [25], GSTM2 overexpression attenuated IRF3 phosphorylation in PE-treated cardiomyocytes (Fig. 7E) as well as IFN-I production (Fig. 7F). In addition, blocking IFNAR1 with an anti-IFNAR1 antibody significantly inhibited the macrophage inflammation that was induced by culture supernatants from PE-treated cardiomyocytes (Fig. 7G). Collectively, these results indicate that GSTM2 inhibits cardiomyocyte eccDNA and IFN-I release, which causes macrophage inflammation during the progression of cardiac hypertrophy.

## Discussion

HF seriously threatens people’s lives worldwide [28]. Recently, a proteomic map of the healthy human heart was established as a reference for comparison against footprints of malfunctioning hearts [11]. In this study, we analyzed the proteomics and transcriptomics of healthy and hypertrophic human hearts. Importantly, our study shows that there are substantial differences in changes in protein expression between the atria and ventricles of myocardium tissues from patients with HF. Interestingly, the metabolic state was altered in the ventricular tissues from HF patients, and inflammatory pathways were activated in atrial tissues. In addition, we found that GSTM2, which is a detoxification enzymes, was significantly decreased in HF samples, and GSTM2 in cardiomyocytes inhibited DNA damage and eccDNA production, thereby alleviating macrophage activation and inflammation and eventually ameliorating cardiac hypertrophy. Our study provides a framework for the deeper interrogation of proteomics and transcriptomics of HF in humans, and highlights the functional importance of GSTM2 in the heart, and provides a novel therapeutic target for HF.

The atria are mainly responsible for collecting and transferring pulmonary and systemic blood, and ventricles are responsible for pumping blood throughout the entire body [29]. Consistent with their functions, mitochondrial proteins and lipid metabolism-related proteins were more abundant in healthy ventricles than in atria [11]. An increasing number of studies have reported that immune activation and inflammation are considered important drivers of cardiac remodeling and HF [30, 31]. Interestingly, we found that the proteins that were specifically upregulated in the atria of HF samples were mainly enriched in the complement and coagulation cascades, which cause overt inflammation, thrombotic microangiopathy and end-organ damage [32–34]; however, the proteins that were specifically upregulated in the ventricles of HF samples were mainly enriched in the FoxO signaling pathway, which is involved in maintaining cardiomyocytes in the homeostatic state and inducing their adaptation to metabolism [35, 36], and the apelin signaling pathway, which plays a critical role in the positive inotropic effect and maintains cardiac contractility [37, 38]. The proteins that were specifically downregulated in the atria of HF samples were mainly enriched in the tight junction and focal adhesion signaling pathways, indicating endothelial dysfunction and cardiac inflammation [39, 40]. These findings suggest that inflammation may occur in the atria in the early stage of HF and that activation of FoxO and apelin signaling pathways in the ventricles represents an attempt to maintain cardiac contractility. Consistent with our speculation, pseudotime alignment of cardiomyocytes with reduced cardiac function (2–5 weeks) from a pressure overload-induced cardiac hypertrophy mouse model exhibited significant enrichment in the immune response and the response to cytokines and chemokines, indicating increased inflammation [41]. Activation of proinflammatory macrophages, which are characterized by abundant expression of inflammatory markers, is a key event in the middle stage of cardiac hypertrophy; inhibition of macrophage activation by dapagliflozin, which a sodium glucose cotransporter 2 inhibitor, in clinical trials for patients with HF as well as by TD139 and arglabin, which are two novel anti-inflammatory agents that are used to treat cardiac diseases, preserves cardiac function and attenuates fibrosis [41]. Thus, alleviation of the inflammatory response is essential for preventing HF in the early stage of cardiac hypertrophy.

GSTs, which are key multifunctional phase II detoxification enzymes, play essential roles in detoxification, metabolism, and prevention of oxidation [42, 43]. Our study found that several GST family members, including MGST1, GSTT1, GSTZ1, and GSTM2, were significantly decreased in the HF samples, and GSTM2 was the most widely distributed in heart tissues. A previous study showed that GSTM2 was significantly reduced in the LV of 16-week-old spontaneously hypertensive rats and functioned to protect cells from OS-associated damage and cell death [44]. GSTM2 efficiently alleviated benzo[a]pyrene-diolepoxide- or benzo[a]pyrene-diolepoxide-induced DNA damage in lung cancer cells [45, 46]. Consistent with a previous study, our study also showed that GSTM2 was reduced in hypertrophic heart tissues from both humans and mice. Furthermore, overexpression of GSTM2 efficiently alleviated cardiac hypertrophy and improved heart function by inhibiting DNA damage, eccDNA production, and IFN-I release by cardiomyocytes and suppressing macrophage inflammation. Interestingly, a similar reduction in GSTM2 was identified in the LVs and aortas of 4-, 8-, and 16-week-old spontaneously hypertensive rats, which develop numerous cardiovascular complications, including cardiac hypertrophy and HF before the onset of hypertension [47, 48]. This suggests that a reduction in GSTM2 in the early stage may be an essential inducer of cardiac hypertrophy and HF. Recently, GSTM2 was shown to be a key molecular determinant of resistance of prostate to several second-generation androgen receptor inhibitors (SG-ARIs), and aryl hydrocarbon receptor (AhR) is the upstream transcription factor that induce the overexpression of GSTM2 in enzalutamide-resistant PCa [49]. However, whether the reduction in GSTM2 in heart tissues during the progression of cardiac hypertrophy and HF is related to AhR and the underlying mechanism need to be further investigated in future studies.

There are still some limitations in the present study. First, the samples that were used for the proteomic and transcriptomic analyses were obtained from only eight HF patients and three healthy donors. More samples need to be collected to further confirm our results in future studies. Second, AAV-mediated GSTM2 overexpression was used to explore the beneficial role of GSTM2 in TAC model mice. Mice with cardiomyocyte-specific GSTM2 overexpression should be constructed and used in future studies.

In conclusion, our study establishes a proteomic and transcriptomic map of human HF tissues, highlights the functional importance of GSTM2 in HF progression, and elucidates potential new targets for treatment of cardiac hypertrophy and HF.

### Supplementary Information

**Additional file 1.** Baseline characteristics of patients.

**Additional file 2.** The supplementray figures for GSTM2 alleviates heart failure by inhibiting DNA damage in cardiomyocytes.

## Data Availability

The mass spectrometry proteomics data have been deposited to the ProteomeXchange Consortium (OMIX004754). The raw sequence data reported in this paper have been deposited in the Genome Sequence Archive (Genomics, Proteomics & Bioinformatics 2021) in National Genomics Data Center (Nucleic Acids Res 2022), China National Center for Bioinformation/Beijing Institute of Genomics, Chinese Academy of Sciences (GSA-Human: HRA005298) that are publicly accessible at https://ngdc.cncb.ac.cn/gsa-human. All data generated and/or analyzed during this study are included in this published article.

## References

[1] Roth GA, Johnson C, Abajobir A, Abd-Allah F, Abera SF, Abyu G, Ahmed M, Aksut B, Alam T, Alam K, Alla F, Alvis-Guzman N, Amrock S, Ansari H, Arnlov J, Asayesh H, Atey TM, Avila-Burgos L, Awasthi A, Banerjee A, Barac A, Barnighausen T, Barregard L, Bedi N, Belay Ketema E, Bennett D, Berhe G, Bhutta Z, Bitew S, Carapetis J, Carrero JJ, Malta DC, Castaneda-Orjuela CA, Castillo-Rivas J, Catala-Lopez F, Choi JY, Christensen H, Cirillo M, Cooper L, Criqui M, Cundiff D, Damasceno A, Dandona L, Dandona R, Davletov K, Dharmaratne S, Dorairaj P, Dubey M, Ehrenkranz R, El Sayed Zaki M, Faraon EJA, Esteghamati A, Farid T, Farvid M, Feigin V, Ding EL, Fowkes G, Gebrehiwot T, Gillum R, Gold A, Gona P, Gupta R, Habtewold TD, Hafezi-Nejad N, Hailu T, Hailu GB, Hankey G, Hassen HY, Abate KH, Havmoeller R, Hay SI, Horino M, Hotez PJ, Jacobsen K, James S, Javanbakht M, Jeemon P, John D, Jonas J, Kalkonde Y, Karimkhani C, Kasaeian A, Khader Y, Khan A, Khang YH, Khera S, Khoja AT, Khubchandani J, Kim D, Kolte D, Kosen S, Krohn KJ, Kumar GA, Kwan GF, Lal DK, Larsson A, Linn S, Lopez A, Lotufo PA, El Razek HMA (2017). Global, regional, and national burden of cardiovascular diseases for 10 causes, 1990 to 2015. J Am Coll Cardiol.

[2] Cvijic M, Rib Y, Danojevic S, Radulescu CI, Nazghaidze N, Vardas P (2022). Heart failure with mildly reduced ejection fraction: from diagnosis to treatment. Gaps and dilemmas in current clinical practice. Heart Fail Rev.

[3] Hagendorff A, Helfen A, Brandt R, Altiok E, Breithardt O, Haghi D, Knierim J, Lavall D, Merke N, Sinning C, Stobe S, Tschope C, Knebel F, Ewen S (2022). Expert proposal to characterize cardiac diseases with normal or preserved left ventricular ejection fraction and symptoms of heart failure by comprehensive echocardiography. Clin Res Cardiol.

[4] Yang D, Liu HQ, Liu FY, Guo Z, An P, Wang MY, Yang Z, Fan D, Tang QZ (2021). Mitochondria in pathological cardiac hypertrophy research and therapy. Front Cardiovasc Med.

[5] Velcea AE, Mihaila Baldea S, Nicula AI, Vinereanu D (2022). The role of multimodality imaging in the selection for implantable cardioverter-defibrillators in heart failure: A narrative review. J Clin Ultrasound.

[6] Guzik M, Urban S, Iwanek G, Biegus J, Ponikowski P, Zymlinski R (2022). Novel therapeutic devices in heart failure. J Clin Med.

[7] Anderson ME, Brown JH, Bers DM (2011). CaMKII in myocardial hypertrophy and heart failure. J Mol Cell Cardiol.

[8] Heineke J, Molkentin JD (2006). Regulation of cardiac hypertrophy by intracellular signalling pathways. Nat Rev Mol Cell Biol.

[9] Csardi G, Franks A, Choi DS, Airoldi EM, Drummond DA (2015). Accounting for experimental noise reveals that mRNA levels, amplified by post-transcriptional processes, largely determine steady-state protein levels in yeast. PLoS Genet.

[10] Lau E, Cao Q, Lam MPY, Wang J, Ng DCM, Bleakley BJ, Lee JM, Liem DA, Wang D, Hermjakob H, Ping P (2018). Integrated omics dissection of proteome dynamics during cardiac remodeling. Nat Commun.

[11] Doll S, Dressen M, Geyer PE, Itzhak DN, Braun C, Doppler SA, Meier F, Deutsch MA, Lahm H, Lange R, Krane M, Mann M (2017). Region and cell-type resolved quantitative proteomic map of the human heart. Nat Commun.

[12] Zhang Q, Lou Y, Yang J, Wang J, Feng J, Zhao Y, Wang L, Huang X, Fu Q, Ye M, Zhang X, Chen Y, Ma C, Ge H, Wang J, Wu J, Wei T, Chen Q, Wu J, Yu C, Xiao Y, Feng X, Guo G, Liang T, Bai X (2019). Integrated multiomic analysis reveals comprehensive tumour heterogeneity and novel immunophenotypic classification in hepatocellular carcinomas. Gut.

[13] Jiang J, Zhao J, Liu D, Zhang M (2022). Different roles of urinary light chains and serum light chains as potential biomarkers for monitoring disease activity in systemic lupus erythematosus. PeerJ.

[14] Yang N, Wang W, Wen R, Zhang TN, Liu CF (2022). Integrated insights into the mechanisms underlying sepsis-induced myocardial depression using a quantitative global proteomic analysis. J Proteomics.

[15] Yu G, Wang LG, Han Y, He QY (2012). clusterProfiler: an R package for comparing biological themes among gene clusters. OMICS.

[16] Langfelder P, Horvath S (2008). WGCNA: an R package for weighted correlation network analysis. BMC Bioinformatics.

[17] Xu H, Wang Z, Chen M, Zhao W, Tao T, Ma L, Ni Y, Li W (2021). YTHDF2 alleviates cardiac hypertrophy via regulating Myh7 mRNA decoy. Cell Biosci.

[18] Wang L, Yu P, Zhou B, Song J, Li Z, Zhang M, Guo G, Wang Y, Chen X, Han L, Hu S (2020). Single-cell reconstruction of the adult human heart during heart failure and recovery reveals the cellular landscape underlying cardiac function. Nat Cell Biol.

[19] Goldenberg JR, Carley AN, Ji R, Zhang X, Fasano M, Schulze PC, Lewandowski ED (2019). Preservation of acyl coenzyme a attenuates pathological and metabolic cardiac remodeling through selective lipid trafficking. Circulation.

[20] Bogomolovas J, Gasch A, Bajoras V, Karciauskaite D, Serpytis P, Grabauskiene V, Labeit D, Labeit S (2016). Cardiac specific titin N2B exon is a novel sensitive serological marker for cardiac injury. Int J Cardiol.

[21] Andra K, Lassmann H, Bittner R, Shorny S, Fassler R, Propst F, Wiche G (1997). Targeted inactivation of plectin reveals essential function in maintaining the integrity of skin, muscle, and heart cytoarchitecture. Genes Dev.

[22] Yuan ZY, Cheng LT, Wang ZF, Wu YQ (2021). Desmoplakin and clinical manifestations of desmoplakin cardiomyopathy. Chin Med J (Engl).

[23] Fafian-Labora JA, Rodriguez-Navarro JA, Ologhlen A (2020). Small extracellular vesicles have GST activity and ameliorate senescence-related tissue damage. Cell Metab.

[24] Turnu L, Di Minno A, Porro B, Squellerio I, Bonomi A, Manega CM, Werba JP, Parolari A, Tremoli E, Cavalca V (2017). Assessing free-radical-mediated DNA damage during cardiac surgery: 8-oxo-7,8-dihydro-2'-deoxyguanosine as a putative biomarker. Oxid Med Cell Longev.

[25] Wang Y, Wang M, Djekidel MN, Chen H, Liu D, Alt FW, Zhang Y (2021). eccDNAs are apoptotic products with high innate immunostimulatory activity. Nature.

[26] Dillon LW, Kumar P, Shibata Y, Wang YH, Willcox S, Griffith JD, Pommier Y, Takeda S, Dutta A (2015). Production of extrachromosomal MicroDNAs is linked to mismatch repair pathways and transcriptional activity. Cell Rep.

[27] Shemesh M, Lochte S, Piehler J, Schreiber G (2021). IFNAR1 and IFNAR2 play distinct roles in initiating type I interferon-induced JAK-STAT signaling and activating STATs. Sci Signal.

[28] Skalidis I, Lu H, Antiochos P, Pitta Gros B, Auberson D, Domenichini G, Carroz P, Teres C, Messaoudi Y, Fournier S, Rutz T, Bouchardy J, Pascale P, Monney P, Hullin R, Eeckhout E, Schwitter J, Pruvot E, Muller O (2023). Cardiology: what’s new in 2022. Rev Med Suisse.

[29] Cheng HM (2022). Emerging MRI techniques for molecular and functional phenotyping of the diseased heart. Front Cardiovasc Med.

[30] Wienecke LM, Leid JM, Leuschner F, Lavine KJ (2023). Imaging targets to visualize the cardiac immune landscape in heart failure. Circ Cardiovasc Imaging.

[31] Noutsias M, Rohde M, Goldner K, Block A, Blunert K, Hemaidan L, Hummel M, Blohm JH, Lassner D, Kuhl U, Schultheiss HP, Volk HD, Kotsch K (2011). Expression of functional T-cell markers and T-cell receptor Vbeta repertoire in endomyocardial biopsies from patients presenting with acute myocarditis and dilated cardiomyopathy. Eur J Heart Fail.

[32] Zabczyk M, Ariens RAS, Undas A (2023). Fibrin clot properties in cardiovascular disease: from basic mechanisms to clinical practice. Cardiovasc Res.

[33] Oikonomopoulou K, Ricklin D, Ward PA, Lambris JD (2012). Interactions between coagulation and complement–their role in inflammation. Semin Immunopathol.

[34] Kornej J, Buttner P, Hammer E, Engelmann B, Dinov B, Sommer P, Husser D, Hindricks G, Volker U, Bollmann A (2018). Circulating proteomic patterns in AF related left atrial remodeling indicate involvement of coagulation and complement cascade. PLoS ONE.

[35] Ni YG, Wang N, Cao DJ, Sachan N, Morris DJ, Gerard RD, Kuro OM, Rothermel BA, Hill JA (2007). FoxO transcription factors activate Akt and attenuate insulin signaling in heart by inhibiting protein phosphatases. Proc Natl Acad Sci U S A.

[36] Bugger H, Abel ED (2014). Molecular mechanisms of diabetic cardiomyopathy. Diabetologia.

[37] Japp AG, Cruden NL, Barnes G, van Gemeren N, Mathews J, Adamson J, Johnston NR, Denvir MA, Megson IL, Flapan AD, Newby DE (2010). Acute cardiovascular effects of apelin in humans: potential role in patients with chronic heart failure. Circulation.

[38] Szokodi I, Tavi P, Foldes G, Voutilainen-Myllyla S, Ilves M, Tokola H, Pikkarainen S, Piuhola J, Rysa J, Toth M, Ruskoaho H (2002). Apelin, the novel endogenous ligand of the orphan receptor APJ, regulates cardiac contractility. Circ Res.

[39] Abelanet A, Camoin M, Rubin S, Bougaran P, Delobel V, Pernot M, Forfar I, Guilbeau-Frugier C, Gales C, Bats ML, Renault MA, Dufourcq P, Couffinhal T, Duplaa C (2022). Increased capillary permeability in heart induces diastolic dysfunction independently of inflammation, fibrosis, or cardiomyocyte dysfunction. Arterioscler Thromb Vasc Biol.

[40] Murphy JM, Jeong K, Cioffi DL, Campbell PM, Jo H, Ahn EE, Lim SS (2021). Focal adhesion kinase activity and localization is critical for TNF-alpha-induced nuclear factor-kappaB activation. Inflammation.

[41] Ren Z, Yu P, Li D, Li Z, Liao Y, Wang Y, Zhou B, Wang L (2020). Single-cell reconstruction of progression trajectory reveals intervention principles in pathological cardiac hypertrophy. Circulation.

[42] Nakanishi G, Pita-Oliveira M, Bertagnolli LS, Torres-Loureiro S, Scudeler MM, Cirino HS, Chaves ML, Miwa B, Rodrigues-Soares F (2022). Worldwide systematic review of GSTM1 and GSTT1 null genotypes by continent, ethnicity, and therapeutic area. OMICS.

[43] Sreekumar PG, Ferrington DA, Kannan R (2021). Glutathione metabolism and the novel role of mitochondrial GSH in retinal degeneration. Antioxidants (Basel).

[44] Shah AK, Bhullar SK, Elimban V, Dhalla NS (2021). Oxidative stress as a mechanism for functional alterations in cardiac hypertrophy and heart failure. Antioxidants (Basel).

[45] Goncharova NS, Moiseeva OM, Shliakhto EV, Aleshina GM (2007). Matrix metalloproteinases: significance in remodeling of the myocardium in valvular heart disease. Kardiologiia.

[46] Ayoub KF, Pothineni NVK, Rutland J, Ding Z, Mehta JL (2017). Immunity, inflammation, and oxidative stress in heart failure: emerging molecular targets. Cardiovasc Drugs Ther.

[47] Rysa J, Leskinen H, Ilves M, Ruskoaho H (2005). Distinct upregulation of extracellular matrix genes in transition from hypertrophy to hypertensive heart failure. Hypertension.

[48] Zhou SG, Wang P, Pi RB, Gao J, Fu JJ, Fang J, Qin J, Zhang HJ, Li RF, Chen SR, Tang FT, Liu PQ (2008). Reduced expression of GSTM2 and increased oxidative stress in spontaneously hypertensive rat. Mol Cell Biochem.

[49] Li C, Liu J, He D, Mao F, Rao X, Zhao Y, Lanman NA, Kazemian M, Farah E, Liu J, Ngule CM, Zhang Z, Zhang Y, Kong Y, Li L, Wang C, Liu X (2022). GSTM2 is a key molecular determinant of resistance to SG-ARIs. Oncogene.

