# Supplementary material for: GSTM2 alleviates heart failure by inhibiting DNA damage in cardiomyocytes
Source: Cell Biosci. 2023 Nov 30;13:220. doi: 10.1186/s13578-023-01168-3 (PMC10688053; doi:10.1186/s13578-023-01168-3)
Supplement: Supplementary file 2 — Additional file 2. The supplementray figures for GSTM2 alleviates heart failure by inhibiting DNA damage in cardiomyocytes. [file 13578_2023_1168_MOESM2_ESM.doc]

Figure S1

Heatmap of the proteins identified by proteomics in different samples. (B and C) Gene count distribution in different patient samples or in different regions. (D and E) Enriched gene distribution in different patient samples or in different regions. (F and G) qRT‒PCR analysis of the indicated mRNA expression levels in human HF tissues.

Figure S2

1. Unsupervised hierarchical clustering analysis of the identified genes with significantly different expression across samples (FPKM < 0.05). (B) Coexpression analysis by WGCNA. Heatmap of the genes identified by transcriptomics in different samples. (C) Correlation analysis of the different modules and different heart regions. (D) Correlation analysis between clinical phenotypes and modules.


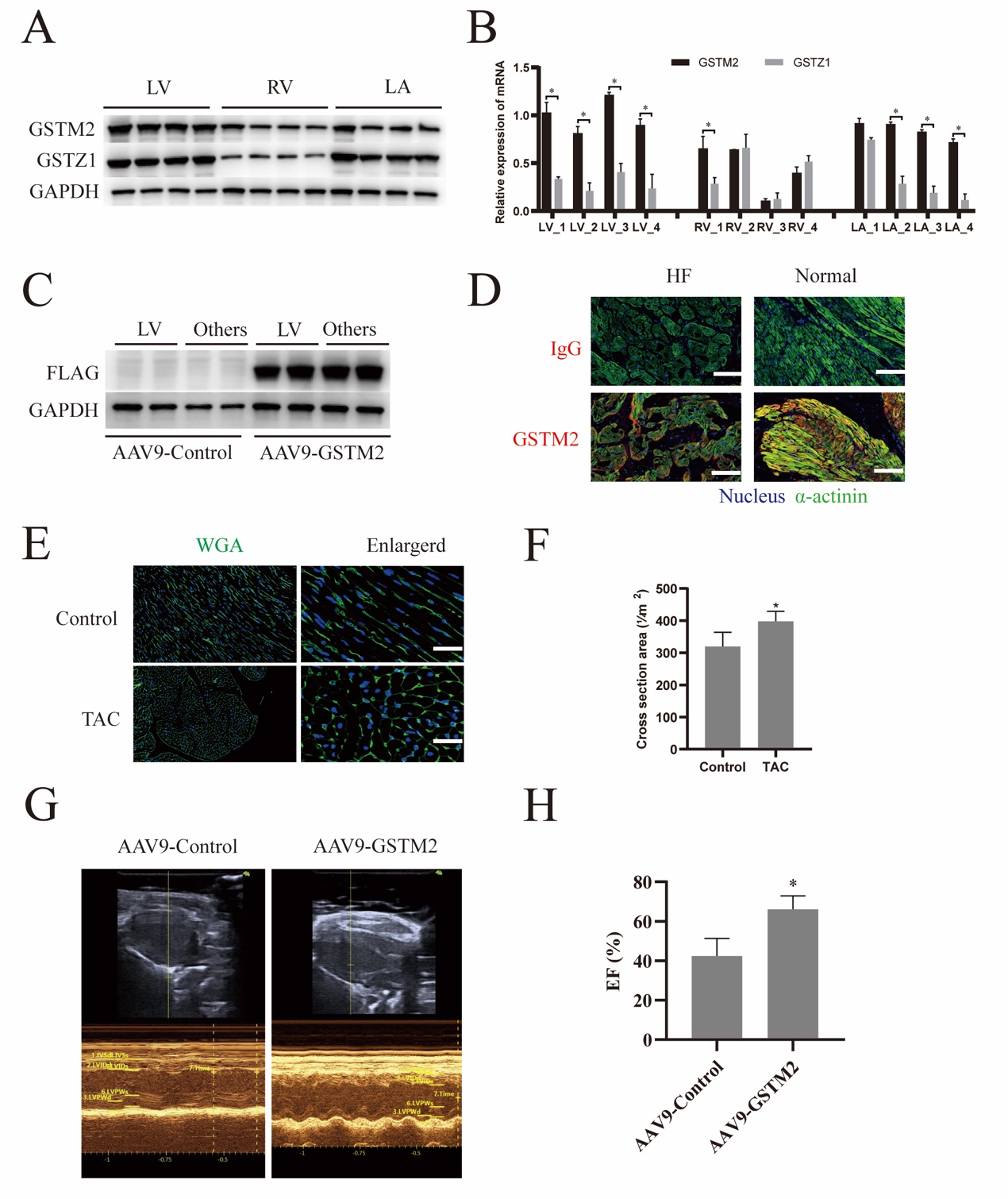


Figure S3

1. Western blotting analysis of GSTM2 and GSTZ1 expression in the indicated regions of human HF tissues. GAPDH was used as a loading control. (B) qRT‒PCR analysis of GSTM2 and GSTZ1 expression in the indicated regions of human HF tissues. (C) Western blotting analysis of flag-GSTM2 expression in the LV region and left heart tissues of TAC model mice infected with AAV9-GSTM2 or control AAV9. GAPDH was used as a loading control. (D) Immunofluorescence analysis of GSTM2 (red) expression in cardiomyocytes (stained with the cardiomyocyte marker α-actinin, green) from clinical human cardiac hypertrophy (CH) tissues and normal heart tissues. The nuclei were stained with DAPI (blue). Scale bar: 250 μm. (E and F) WGA staining (green) was used to measure cardiomyocyte size in the heart tissues of TAC model mice. Statistical analysis of the cross-sectional areas of the indicated groups. Scale bar: 100 μm. (G and H) Representative figures of echocardiographic evaluation of the mouse heart tissues. Statistical analysis of EF (%) of the indicated group. n=6. ^*^*P* < 0.05.
